# Excitatory to inhibitory connectivity shaped by synaptic and homeostatic plasticity

**DOI:** 10.1186/1471-2202-16-S1-P126

**Published:** 2015-12-18

**Authors:** Claudia Clopath, Jacopo Bono, Ulysse Klatzmann

**Affiliations:** 1 Department of Bioengineering, Imperial College London, London, SW7 2AZ, UK

## 

**Figure 1:**
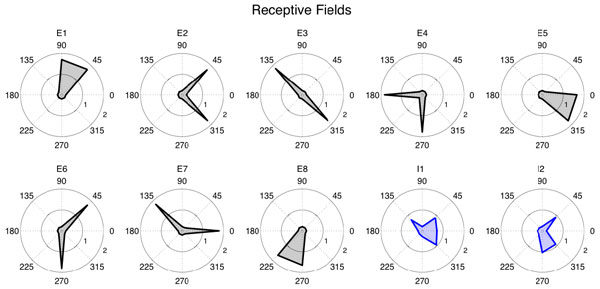
**Orientation preferences of excitatory and inhibitory neurons**. A network of excitatory and inhibitory exponential integrate-and-fire neurons with plastic feedforward inputs, where the recurrent connections from excitatory to excitatory and excitatory to inhibitory connections are plastic under the voltage-triplet STDP rule. After learning, the excitatory neurons which have the same orientation preference have a high chance of being bidirectional connected, as seen in experimentally. On the other hand, inhibitory neurons receive inputs from excitatory neurons with different orientation preferences, consistent with recent experimental results. After learning, receptive fields of the excitatory neurons (black) and the inhibitory neurons (blue). Note that the inhibitory neurons develop broader and more unspecific receptive fields than excitatory neurons.

Recent experimental techniques allowed study of the relationship between neurons' stimulus-preference and connectivity. In particular, in the layer II/III of primary visual cortex, it was shown that excitatory neurons with the same orientation preference have a high probability of being bidirectionally connected. However, the intracortical connectivity is only getting refined after eye-opening. We have recently hypothesized that this process is a result of experience-dependent plasticity, modelled by a Hebbian learning. In contrast to excitatory neurons, parvalbumin-expressing (PV) inhibitory cells are less input-specific: PV neurons receive excitatory inputs from neurons with different orientation preferences. In this work, we investigate the mechanism by which excitatory to inhibitory connections are formed (how) and their potential function (why) in a small recurrent network. We found that a model combining Hebbian learning with homeostatic plasticity, which allows PV neurons to spike at a high rate (i.e reproducing the fast-spiking intrinsic property of the cells), develops unspecific excitatory-to-inhibitory connections (Figure 1). We then tested the role of inhibition by simulating our model with and without inhibition after learning convergence. We found that inhibition ensures less fluctuation of the synaptic weights over time, hence stabilizes the network. We therefore propose that unspecific excitatory to PV connections can be a result of the intrinsic homeostatic property of PV neurons, and can allow the network to be more stable.

